# Supplementary material for: Colloid driven low supersaturation crystallization for atomically thin Bismuth halide perovskite
Source: Nat Commun. 2023 Jun 23;14:3764. doi: 10.1038/s41467-023-39445-x (PMC10290062; doi:10.1038/s41467-023-39445-x)
Supplement: Supplementary file 1 — Supplementary Information [file 41467_2023_39445_MOESM1_ESM.pdf]

Supplementary Information

Colloid Driven Low Supersaturation Crystallization for Atomically Thin Bismuth Halide Perovskite

Li et al.

Contents

Supplementary Figures

Supplementary Fig. 1 The crystal structure of $Cs_3Bi_2Br_9$ and random crystallized $Cs_3Bi_2Br_9$.
Supplementary Fig. 2 The Cs and Bi ions concentration by ICP measurement.
Supplementary Fig. 3 The structure of the colloidal solution.
Supplementary Fig. 4 XPS spectra of the $Cs_3Bi_2Br_9$ that synthesis with and without n-OA.
Supplementary Fig. 5 Infrared spectra of n-OA, $Cs_3Bi_2Br_9$ with and without n-OA.
Supplementary Fig. 6 AFM image of $Cs_3Bi_2Br_9$ on SiO_2/Si substrate.
Supplementary Fig. 7 The representative reports of atomically thin halide perovskite single crystals.
Supplementary Fig. 8 The atomically thin $Cs_3Bi_2Br_9$ growing on untreated or ozone-treated on mica, SiO_2, Si and sapphire substrates.
Supplementary Fig. 9 The effect of wettability on growing atomically thin $Cs_3Bi_2Br_9$.
Supplementary Fig. 10 The effect of temperature on growing atomically thin $Cs_3Bi_2Br_9$.
Supplementary Fig. 11 The effect of the amount of surfactant on growing atomically thin $Cs_3Bi_2Br_9$.
Supplementary Fig. 12 The effect of the organic acid surfactants on growing atomically thin $Cs_3Bi_2Br_9$.
Supplementary Fig. 13 XRD pattern of the atomically thin $Cs_3Bi_2Br_9$.
Supplementary Fig. 14 Raman spectrum and corresponding mapping pattern of atomically thin $Cs_3Bi_2Br_9$.
Supplementary Fig. 15 SHG measurement of atomically thin $Cs_3Bi_2Br_9$.
Supplementary Fig. 16 AFM image of stacking region.

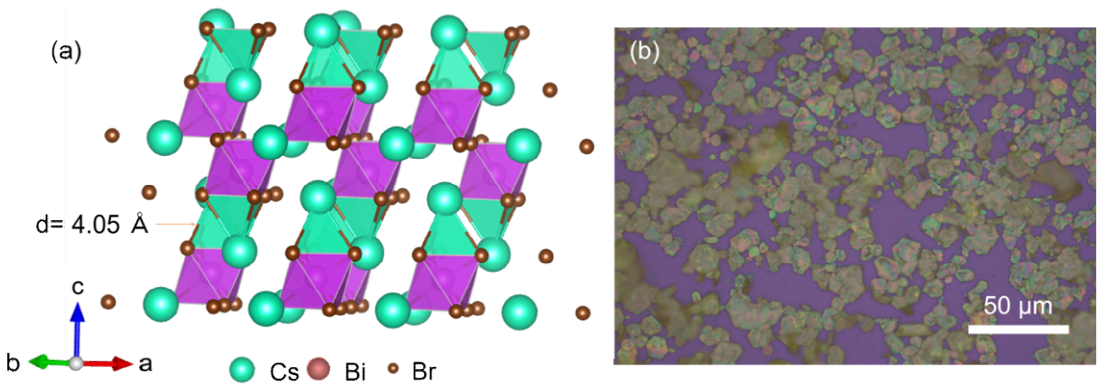

Supplementary Figure 1. **The crystal structure of $Cs_3Bi_2Br_9$ and random crystallized $Cs_3Bi_2Br_9$.** (a) The crystal structure of $Cs_3Bi_2Br_9$ in side view. (b) The production of $Cs_3Bi_2Br_9$ with random crystallization.

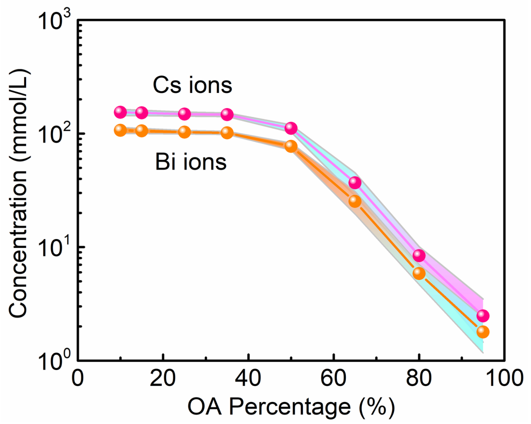

Supplementary Figure 2. **The Cs and Bi ions concentration by ICP measurement.** The Cs and Bi ions concentration of supersaturation state as a function of the n-OA variation. The concentration error bars represent the standard deviation. Source data are provided as a Source Data file.

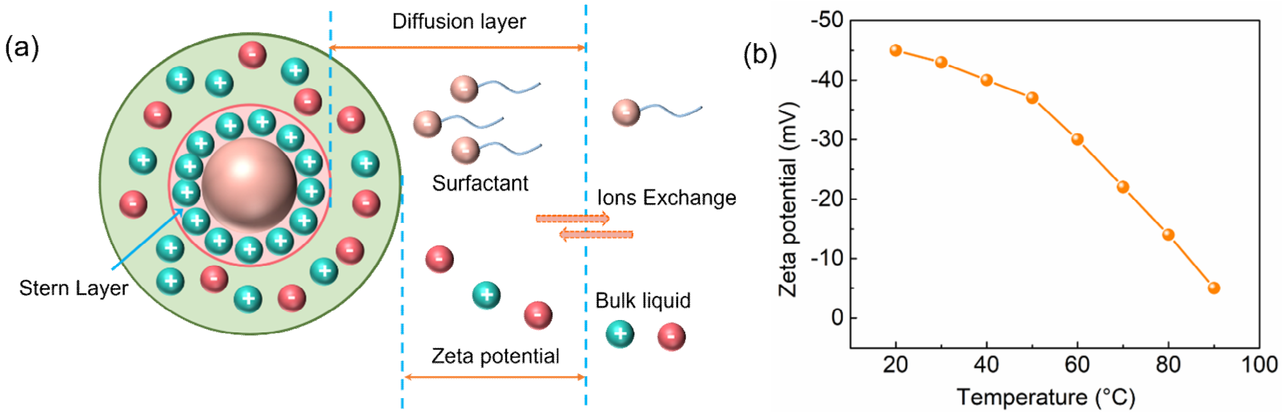

Supplementary Figure 3. **The structure of the colloidal solution.** (a) The Stern double layer model of the colloidal solution. (b) The zeta potential of $Cs_3Bi_2Br_9$ colloidal solution with varied temperature from 20°C to 90°C. The nucleus particle could be negatively charged, attracting positive ions to form the Stern layer, where ions are strongly bound to nucleus particle. The diffusion layer contains a large amount of solvent and ions, and the ions are loosely attached the Stern layer. We carried out the zeta potential of $Cs_3Bi_2Br_9$ colloidal solution to understand the content and mechanism of colloidal solution during growth. While temperature increases, the ions source of colloidal solution continually release for the growth of the atomically thin $Cs_3Bi_2Br_9$ on the substrate with the help of surfactants. Source data are provided as a Source Data file.

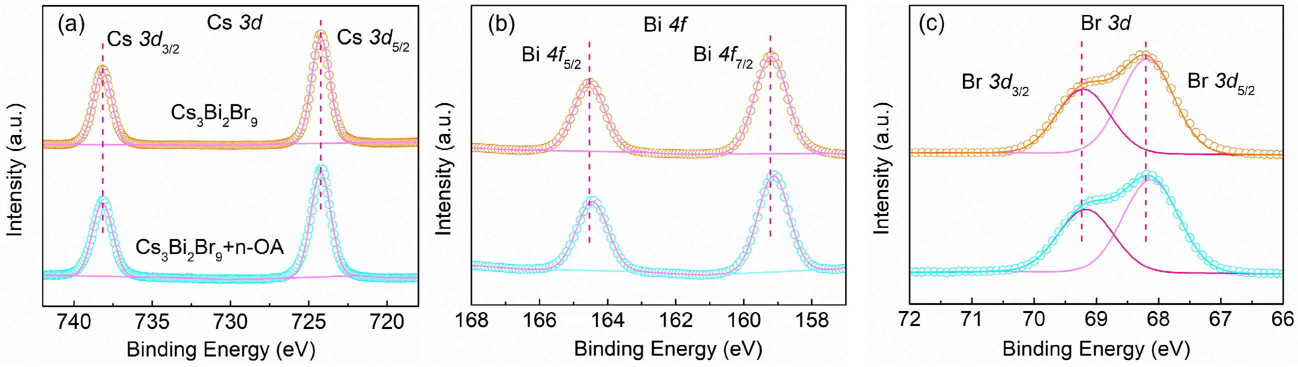

Supplementary Figure 4. **XPS spectra of the $Cs_3Bi_2Br_9$ that synthesis with and without n-OA.** XPS (a) Cs 3*d*, (b) Bi 4*f* and (c) Br 3*d* core level patterns of the $Cs_3Bi_2Br_9$ that synthesis with and without n-OA. Figure S4 shows the XPS of Cs 3*d*, Bi 4*f* and Br 3*d* spectra. There is no evident shift in the Cs 3d peak (Figure S4a). Compared with $Cs_3Bi_2Br_9$, $Cs_3Bi_2Br_9$+n-OA sample shows that core-level peaks of Bi 4*f* (Figure S4b) and Br 3*d* (Figure S4c) are shifted towards low binding energy. This demonstrates that the C=O moiety donates its lone electron pair on the oxygen atoms to the empty 6*p* orbital of Bi^{3+}, not only decreasing the cationic charge but also leading to a change in the electrostatic interaction between the Bi^{3+} and the Br^- ions. Source data are provided as a Source Data file.

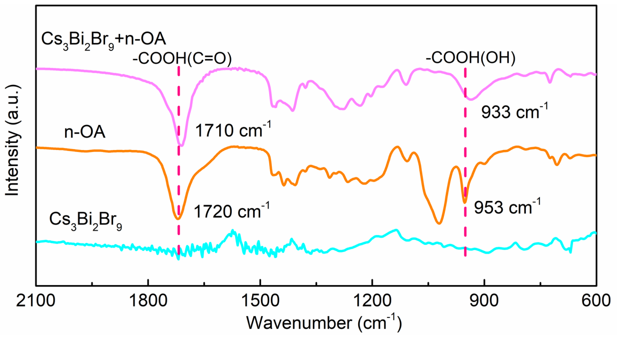

Supplementary Figure 5. **Infrared spectra of n-OA, $Cs_3Bi_2Br_9$ with and without n-OA.** Infrared spectra from 2000 cm^{-1} to 500 cm^{-1} of n-OA, $Cs_3Bi_2Br_9$ with and without n-OA. In the FTIR spectra, we observed an infrared peak at 1720 cm^{-1} arising from the C=O of -COOH group stretching vibration for n-OA. The C=O peak shifts to a lower wavenumber of 1710 cm^{-1} for $Cs_3Bi_2Br_9$+n-OA sample, indicating a weakened C=O bond strength caused by the interaction (Figure S3). The -OH of -COOH group rocking vibration for n-OA is found that this shifted from 953 cm^{-1} to a lower wavenumber 933 cm^{-1} for $Cs_3Bi_2Br_9$+n-OA sample, indicating that there is a slight interaction between n-OA and $Cs_3Bi_2Br_9$ on -COOH group. Source data are provided as a Source Data file.

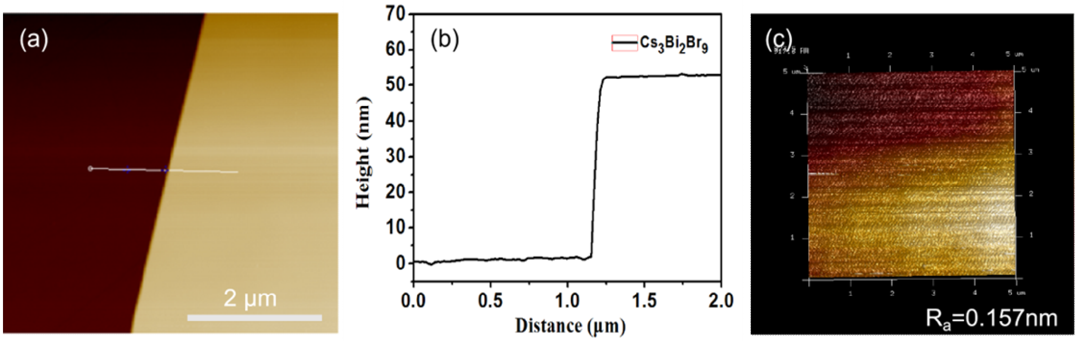

Supplementary Figure 6. **AFM image of $Cs_3Bi_2Br_9$ on SiO_2/Si substrate.** (a) Atomic force microscopy image of the atomically thin $Cs_3Bi_2Br_9$. (b) The corresponding height image that in a. (c) The surface roughness image with an area of 5×5 μm^2 in a.

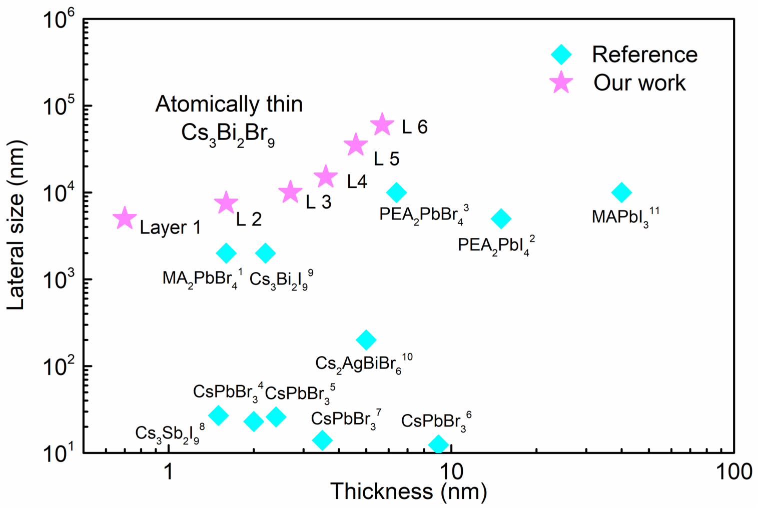

Supplementary Figure 7. **The representative reports of atomically thin halide perovskite single crystals** (The references are shown in **Supplementary References**). Source data are provided as a Source Data file.

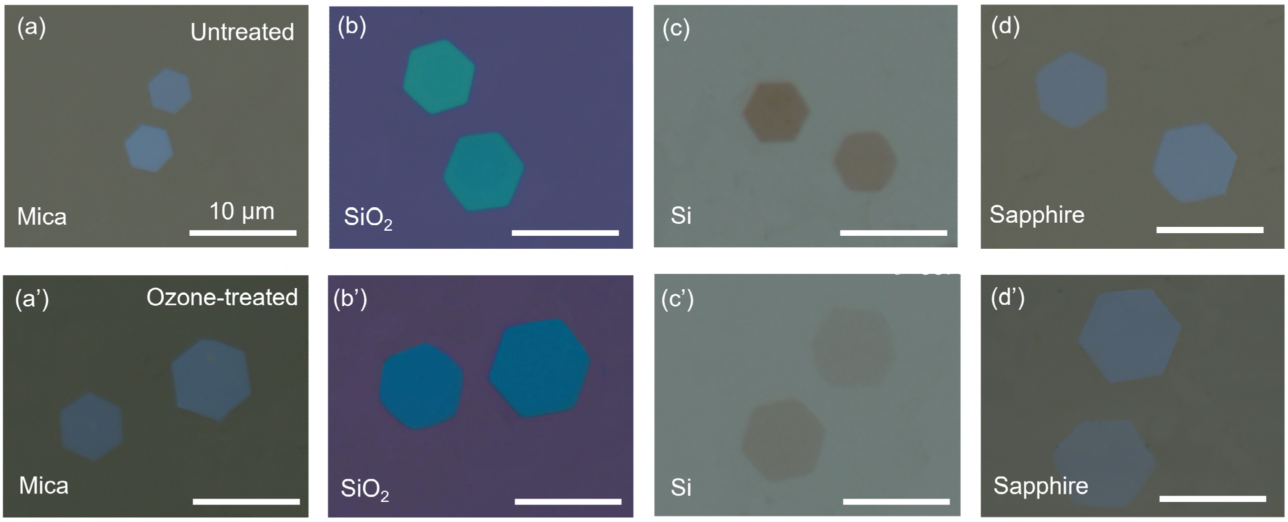

Supplementary Figure 8. **The atomically thin $Cs_3Bi_2Br_9$ growing on untreated or ozone-treated on mica, SiO_2, Si and sapphire substrates.** The atomically thin $Cs_3Bi_2Br_9$ growing on untreated (a) mica, (b) SiO_2, (c) Si and (d) sapphire substrates. The atomically thin $Cs_3Bi_2Br_9$ growing on ozone-treated (a') mica, (b') SiO_2, (c') Si and (d') sapphire substrates.

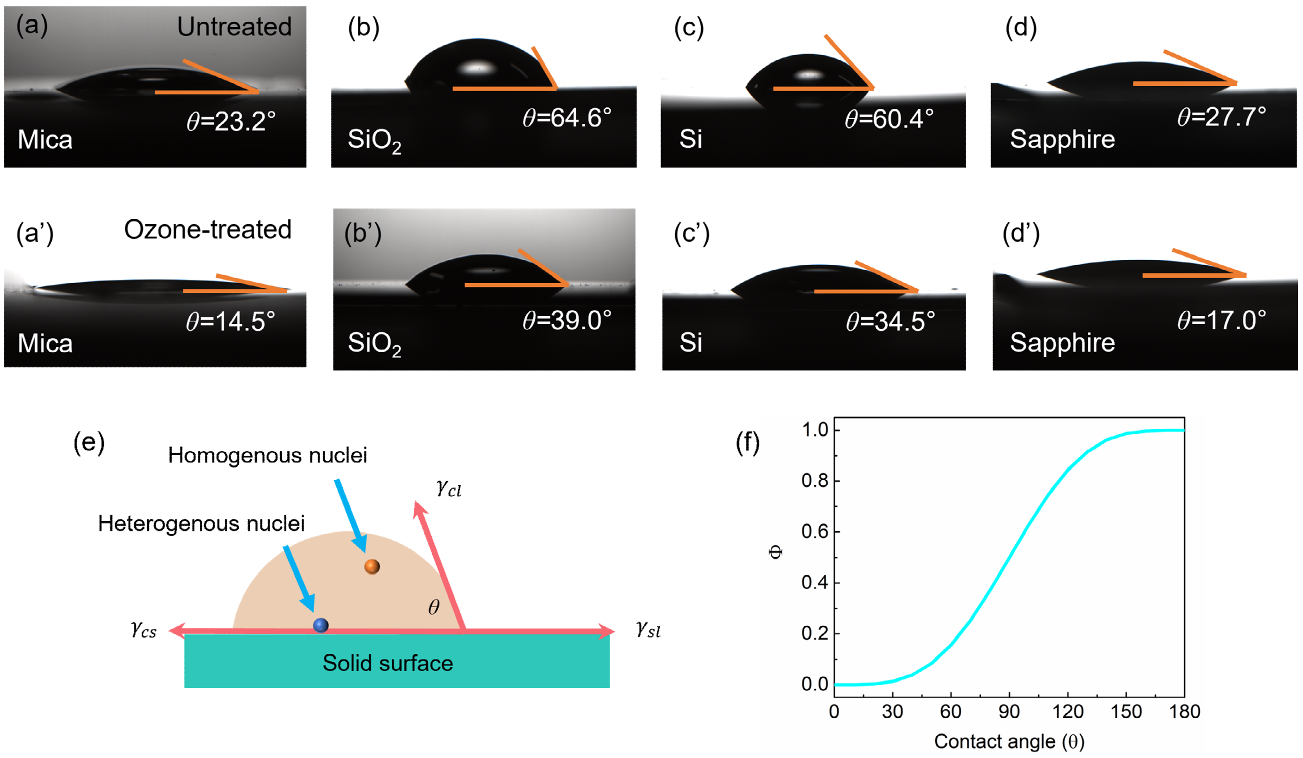

Supplementary Figure 9. **The effect of wettability on growing atomically thin $Cs_3Bi_2Br_9$.** The wettability of the untreated (a) mica, (b) SiO_2, (c) Si and (d) sapphire substrates. The wettability of the ozone-treated (a') mica, (b') SiO_2, (c') Si and (d') sapphire substrates. (e) Illustration of the contact angle θ for heterogeneous nucleation. (f) Ratio of free energies of homogeneous and heterogeneous nucleation Φ as a function of the contact angle θ.

Figure S9a-d presents the wettability of the untreated mica, SiO_2, Si and sapphire substrates. Obviously, the ozone-treated substrates have smaller contact angles than these of untreated substrates (Figure S9a'-d'). Figure S9e illustrates an interfacial energy diagram for three phases of two solids and a liquid in contact. The terms γ_{cl}, γ_{sl} and γ_{cs} represent interfacial energies between the crystal phase and the liquid, the solid surface and the liquid, and the crystal phase and the solid surface, respectively. The nuclei and active centers have a high affinity with the solid surface. And the nucleation barrier (ΔG_{hetero}) is dramatically lowered due to effective reduction in the interface energy. The free energy for heterogeneous nucleation is corrected by introducing Φ, which is the ratio of free energies of homogeneous and heterogeneous nucleation (Eq. 1). Φ is a factor dependent on the contact angle θ (Eq. R).

$$\Delta G_{hetero} = \Phi \Delta G_{hetero} \tag{1}$$

$$\Phi = \frac{(2+cos\theta)(1-cos\theta)^2}{4} \tag{2}$$

The relationship between Φ and θ indicates that heterogeneous nucleation readily occurs at a low concentration state (Figure S9f). In order to grow atomically thin $Cs_3Bi_2Br_9$ with large aspect ratio, it is important to form heterogeneous nuclei on the substrate rather than homogeneous nucleation. As a result, the hydrophilic substrate enhances heterogeneous nucleation to produce bigger lateral sizes of atomically thin $Cs_3Bi_2Br_9$.

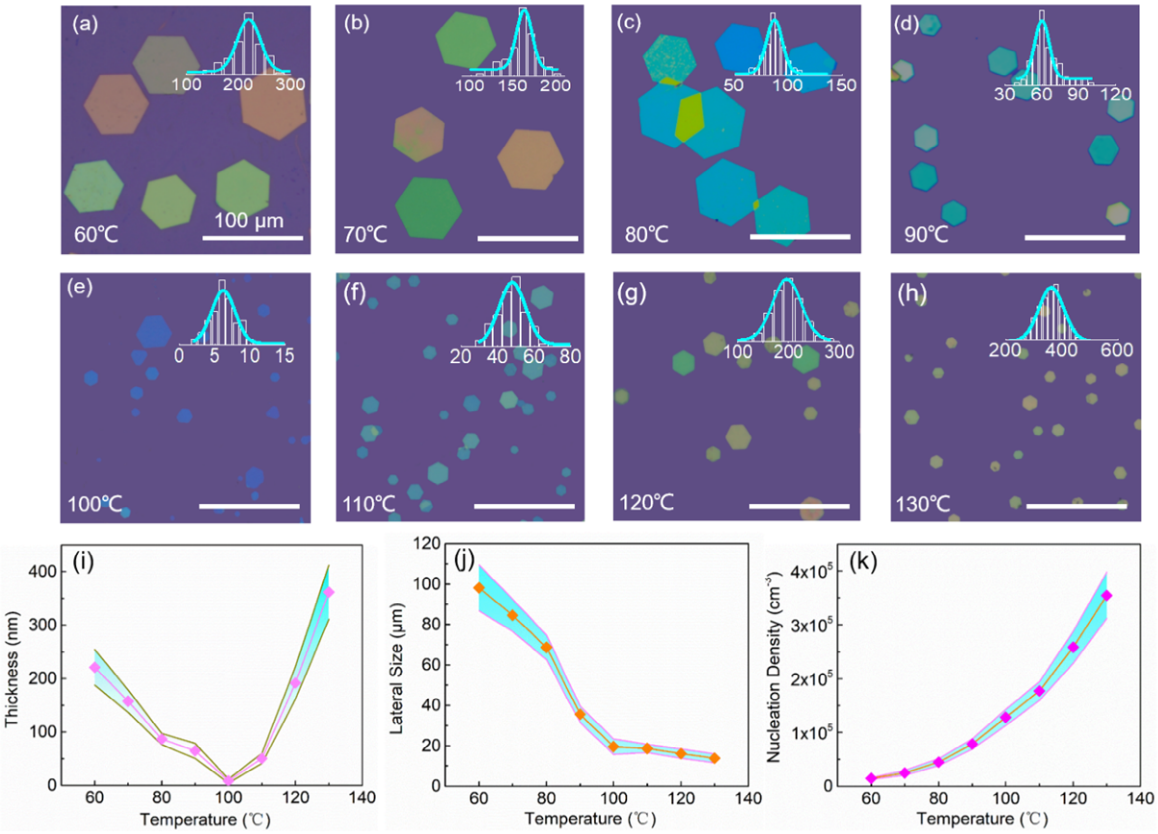

Supplementary Figure 10. **The effect of temperature on growing atomically thin $Cs_3Bi_2Br_9$.** Optical microscope images of the $Cs_3Bi_2Br_9$ that synthesis from ranged temperatures: (a) 60℃, (b) 70℃, (c) 80℃, (d) 90℃, (e) 100℃, (f) 110℃, (g) 120℃ and (h) 130℃, respectively (scale bars, 100 μm). (i) Statistics of the thickness as a function of reaction temperature. The thickness error bars represent the standard deviation. (j) Statistics of the lateral size as a function of reaction temperature. The lateral size error bars represent the standard deviation. (k) Statistics of the nucleation density as a function of reaction temperature. The nucleation density error bars represent the standard deviation. Source data are provided as a Source Data file.

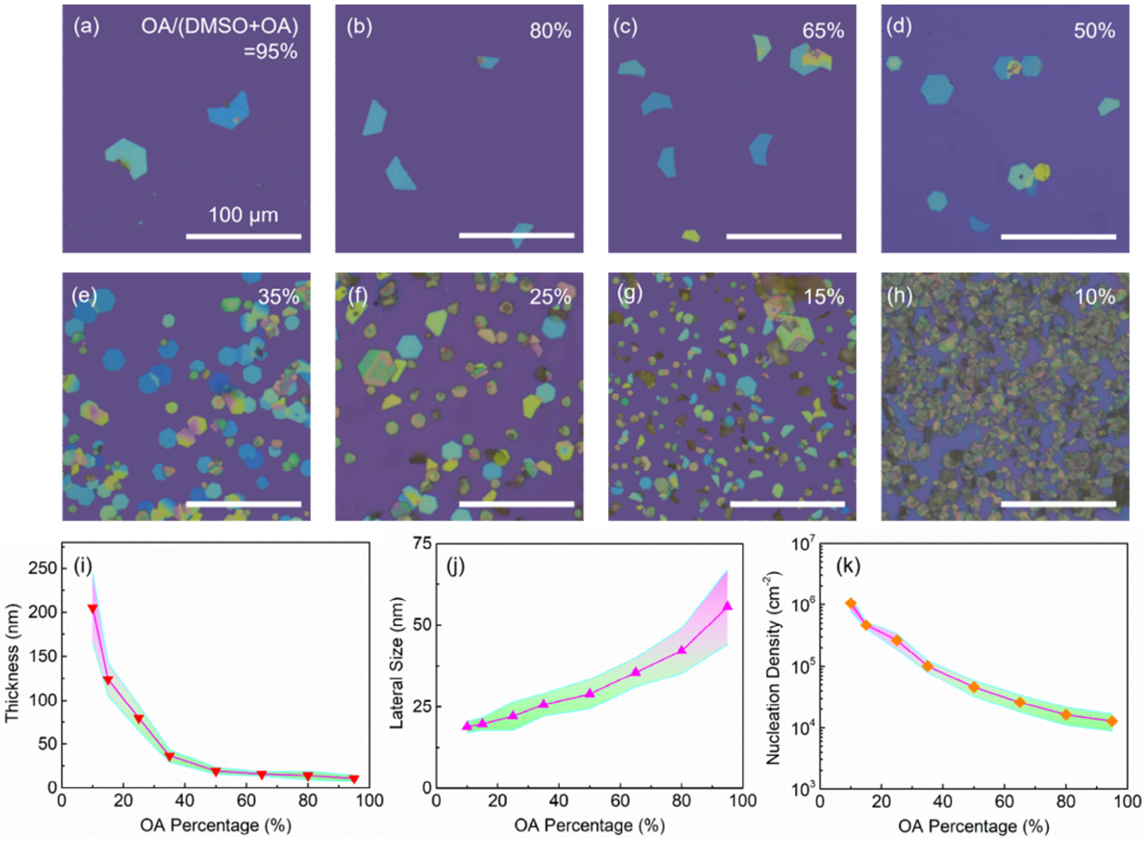

Supplementary Figure 11. **The effect of the amount of surfactant on growing atomically thin $Cs_3Bi_2Br_9$.** Optical microscopy image of the $Cs_3Bi_2Br_9$ that produced from ranged n-OA percentage: (a) 90%, (b) 80%, (c) 65%, (d) 50%, (e) 35%, (f) 25%, (g) 15%, (h) 10%. (i) The statistics of $Cs_3Bi_2Br_9$ thickness as a function of ranged n-OA percentage. (i) The statistics of $Cs_3Bi_2Br_9$ thickness as a function of ranged n-OA percentage. The thickness error bars represent the standard deviation. (g) The statistics of $Cs_3Bi_2Br_9$ lateral size as a function of ranged n-OA percentage. The lateral size error bars represent the standard deviation. (h) The statistics of nucleation density as a function of ranged n-OA percentage. The nucleation density error bars represent the standard deviation. Source data are provided as a Source Data file.

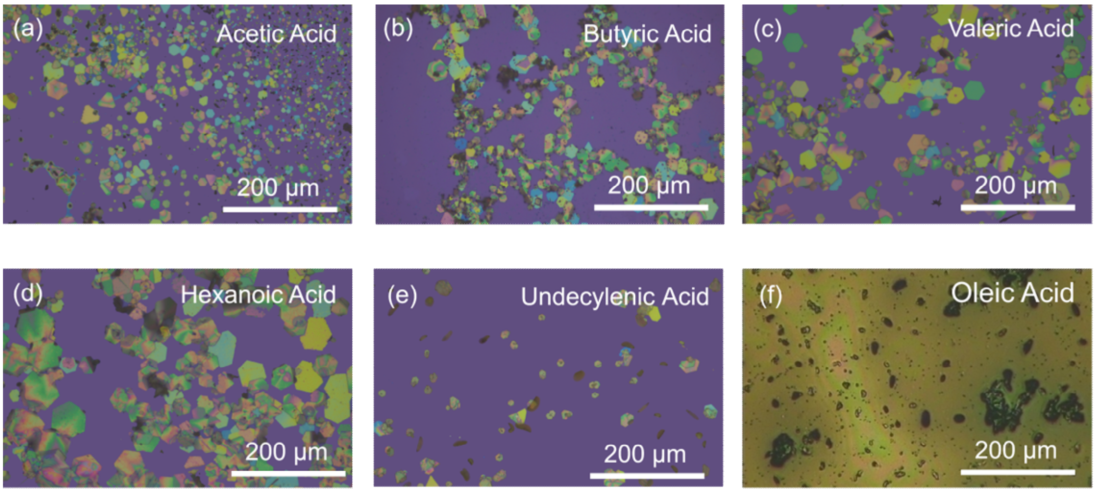

Supplementary Figure 12. **The effect of the organic acid surfactants on growing atomically thin $Cs_3Bi_2Br_9$.** Optical microscopy images of the product that use different organic acid molecular: (a) acetic acid, (b) butyric acid, (c) valeric acid, (d) hexanoic acid, (e) undecylenic acid and (f) oleic acid.

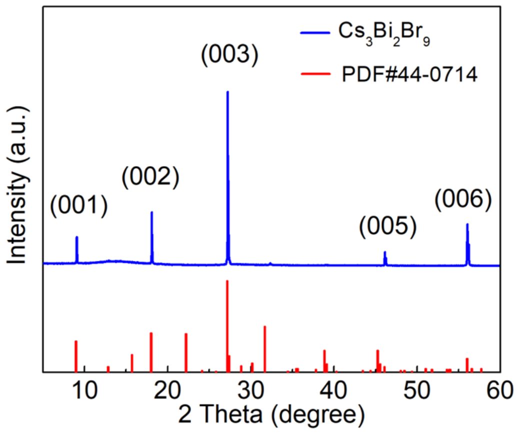

Supplementary Figure 13. **XRD pattern of the atomically thin $Cs_3Bi_2Br_9$.** Source data are provided as a Source Data file.

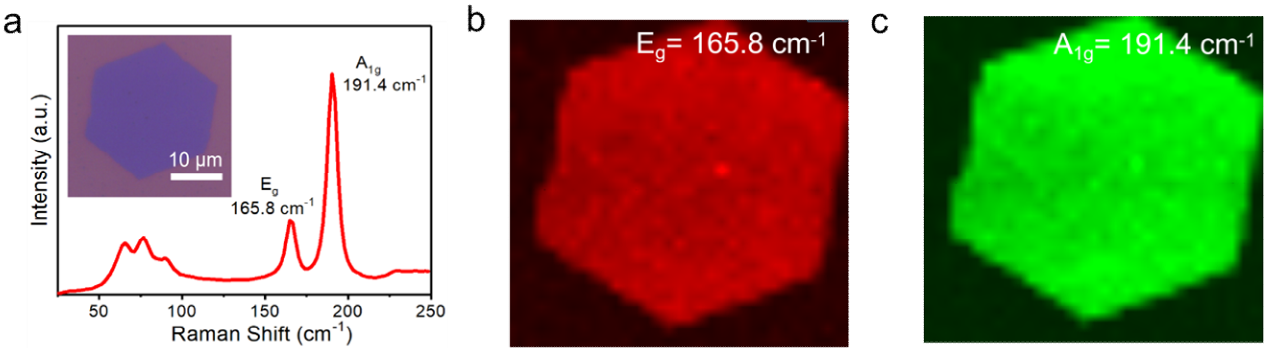

Supplementary Figure 14. **Raman spectrum and corresponding mapping pattern of the atomically thin $Cs_3Bi_2Br_9$.** Source data are provided as a Source Data file.

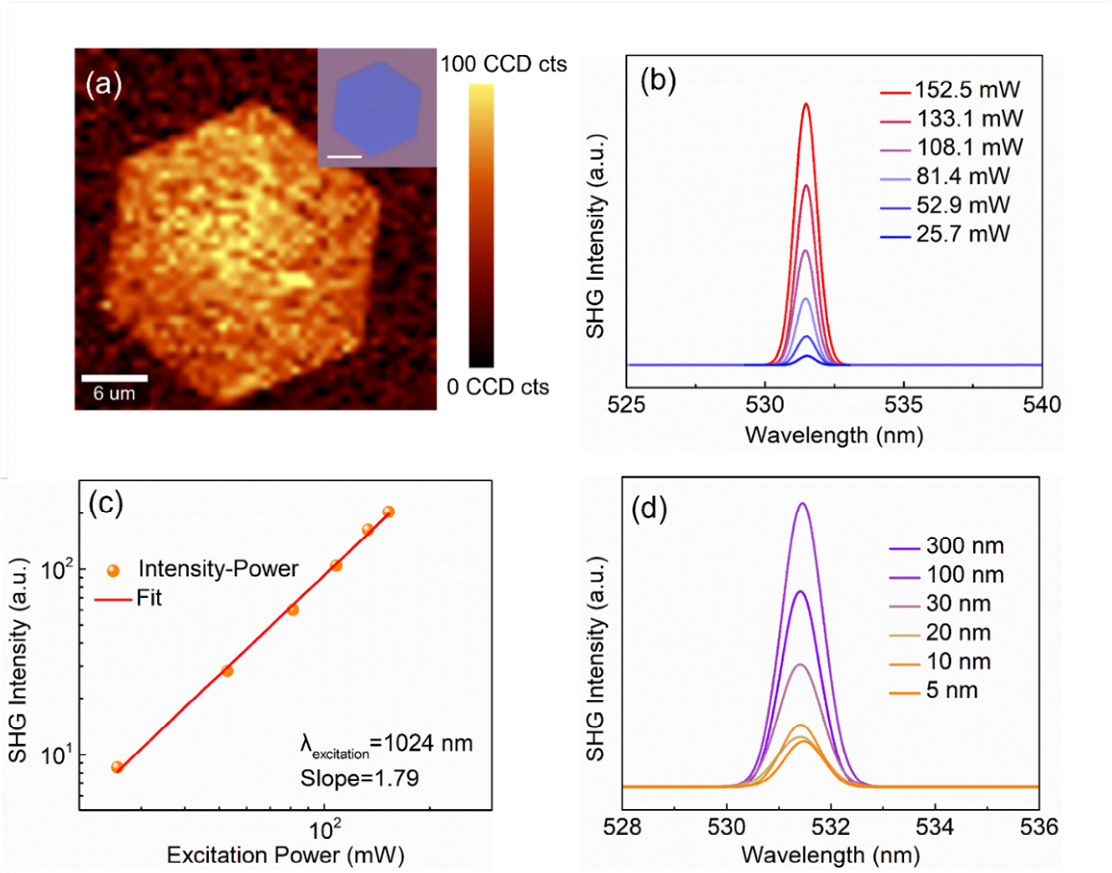

Supplementary Figure 15. **SHG measurement of atomically thin $Cs_3Bi_2Br_9$.** (a) SHG mapping of the atomically thin $Cs_3Bi_2Br_9$. The insert images in the corresponding optical microscope images. Scale bar is 10 μm. (b) Power-dependent SHG spectra of the $Cs_3Bi_2Br_9$. (c) The statistic result of excitation power as a function of SHG intensity and corresponding linear fitting in logarithmic coordinates. (d) Thickness-dependent SHG spectra of the $Cs_3Bi_2Br_9$. Source data are provided as a Source Data file.

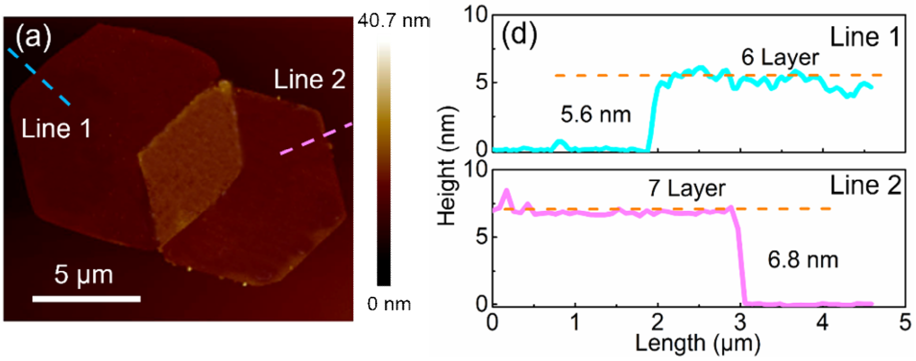

Supplementary Figure 16. **AFM image of stacking region.** (a) The AFM images of the region in Figure 5d. (b) The height of the marked line 1 and line 2 in (a). Source data are provided as a Source Data file.

Supplementary References

1. Dou, L., Wong, A. B., Yu, Y., Lai, M., Kornienko, N., Eaton, S. W., Fu, A. Bischak, C. G., Ma, J., Ding, T., Ginsberg, N. S., Wang, L.-W., Alivisatos, A. P., Yang, P. Atomically thin two-dimensional organic-inorganic hybrid perovskites. *Science* **319**, 1518-1521 (2015).
2. Shi, E., Yuan, B., Shiring, S. B., Gao, Y., Akriti, Guo, Y., Su, C., Lai, M., Yang, P. Two-dimensional halide perovskite lateral epitaxial heterostructures. *Nature* **580**, 614-620 (2020).
3. Akriti, Shi, E., Shiring, S. B., Yang, J., Atencio-Martinez, C. L., Yuan, B., Hu, X., Gao, Y., Finkenauer, B. M., Dou, L. Layer-by-layer anionic diffusion in two-dimensional halide perovskite vertical heterostructures. *Nat. Nanotechnol.* **16**, 584-591 (2021).
4. Yu, W., Li, F., Yu L., Niazi, M. R., Zou, Y., Corzo, D., Basu, A., Ma, C., Dey, S., Tietze, M. L., Buttner, U., Wang, X., Wang, Z., Hedhili, M. N., Guo, C., Wu, T., Amassian, A. Single crystal hybrid perovskite field-effect transistors. *Nat. Commun.* **9**, 5354 (2019).
5. Shamsi, J., Kubocki, D., Anaya, M., Liu, Y., Ji, K., Frohna, K., Grey, C. P., Friend, R. H., Stranks, S. D. Stable hexylphosphonate-capped blue-emitting quantum-confined $CsPbBr_3$ nanoplatelets. *ACS Energy Lett.* **5**, 1900-1907 (2020).
6. Chakrabarty, A., Satija, S., Gangwar, U., Sapra, S. Precursor-mediated synthesis of shape-controlled colloidal $CsPbBr_3$ perovskite nanocrystals and their nanofiber-directed self-assembly. *Chem. Mater.* **32**, 721-733 (2020).
7. Bertolotti, F., Nedelcu, G., Vivani, A., Cervellino, A., Masciocchi, N., Guagliardi, A., Kovalenko, M. V. Crystal structure, morphology, and surface termination of cyan-emissive, six-monolayers-thick $CsPbBr_3$ nanoplatelets from X-ray total scattering. *ACS Nano* **13**, 14294-14307 (2020).
8. Pal, L., Manna, S., Mondal, A., Das, S., Adarsh, K. V., Nag, A. Colloidal synthesis and photophysics of $M_3Sb_2I_9$ (M=Cs and Rb) nanocrystals: Lead-free perovskites. *Angew. Chem. -Int. Ed.* **56**, 14787-14191 (2017).
9. Liang, J., Fang, Q., Wang, H., Xu, R., Jia, S., Guan, Y., Ai, Q., Gao, G., Guo, H., Shen, K., Wen, X., Terlier, T., Wiederrecht, G. P., Qian, X., Zhu, H., Lou, J. Perovskite-derivative valleytronics. *Adv. Mater.* **32**, 2004111 (2020).
10. Huang, J., Zou, S., Lin, J., Liu, Z., Qi, M. Ultrathin lead-free double perovskite cesium silver bismuth bromide nanosheets. *Nano Res.* **14**, 4079-4086 (2021).

11. Dahlman, C. J., Venkatesan, N. R., Corona, P. T., Kennard, R. M., Mao, L., Smith, N. C., Zhang, J., Seshadri, R., Helgeson, M. E., Chabinyc, M. L. Structural evolution of layered hybrid lead iodide perovskites in colloidal dispersions. *ACS Nano* **14**, 11294-11308 (2020).
